# Cancer incidence among Finnish male cobalt production workers in 1969–2013: a cohort study

**DOI:** 10.1186/s12885-017-3333-2

**Published:** 2017-05-18

**Authors:** Riitta Sauni, Panu Oksa, Jukka Uitti, Asko Linna, Raimo Kerttula, Eero Pukkala

**Affiliations:** 1 Department for Occupational Safety and Health, Ministry of Social Affairs and Health, P.O. Box 33, FI-00023 Government Tampere, Finland; 2 Clinic of Occupational Medicine, Tampere University Hospital, Tampere, Finland; 3 Finnish Institute of Occupational Health, Tampere, Finland; 4 The Faculty of Medicine and Life Sciences, University of Tampere, Tampere, Finland; 5 Health Centre of Kokkola town, Kokkola, Finland; 6 Occupational Health, Boliden, Kokkola, Finland; 7 Faculty of Social Sciences, University of Tampere, Tampere, Finland; 8 Finnish Cancer Registry, Institute for Statistical and Epidemiological Cancer Research, Helsinki, Finland

**Keywords:** Cobalt, Occupational exposure, Incidence, Cancer

## Abstract

**Background:**

There is inadequate evidence for the carcinogenicity of cobalt and cobalt compounds in humans. Consequently, the International Agency for Research on Cancer (IARC) has evaluated cobalt metal without tungsten carbide as possibly carcinogenic to humans (Group 2B). The aim of the study was to assess the risk of cancer among workers employed in a Finnish cobalt plant since the beginning of production in 1968.

**Methods:**

The study cohort consisted of all males employed by the Finnish cobalt plant for at least a year during 1968–2004. The follow-up for cancer was performed by studying the files of the Finnish Cancer Registry, using personal identity codes as a key. The cohort was divided into subcohorts by exposure levels. Standardised incidence ratios (SIRs) and 95% confidence intervals (95% CIs) were calculated as ratios of the observed numbers of cancer cases and the numbers expected on the basis of incidence rates in the population of the same region.

**Results:**

The follow-up cohort consisted of 995 men with 26,083 person-years. During the follow-up period, 92 cases of cancer were diagnosed (SIR 1.00, 95% CI 0.81–1.22), six of which were lung cancer cases (SIR 0.50; 95% CI 0.18–1.08). The only cancer type with increased incidence was tongue cancer (three cases, SIR 7.39; 95% CI 1.52–21.6). We observed no dose-response effect across the different exposure levels and the incidence of any cancer type.

**Conclusions:**

The results suggest that occupational exposure to cobalt is not associated with an increased overall cancer risk or lung cancer risk among cobalt workers. Because of the small number of cancer cases the results must be interpreted with caution.

## Background

Workers may be exposed to cobalt during the production of cobalt and cobalt salts, in the production of alloys and hard metal, drying agents, pigments and catalysts, and during diamond polishing. World production of refined cobalt has increased steadily over the last decade, due partly to new operations and partly to a net increase in production by established producers [1]. World cobalt mine production in 2001 was 36,700 tons and the cobalt refinery production 38,400 tons [2]. The respective numbers in 2016 were 123,000 tons and 91,300 tons [3]. In the United States, more than a million workers are potentially exposed to cobalt and its compounds [4]. In Finland, about 1500 workers (0.1% of employed people) are estimated to be exposed to cobalt or cobalt compounds at work [5].

Different epidemiological studies provide relatively sparse and contradictory data on the carcinogenicity of cobalt to humans. A Swedish study retrospectively followed a cohort of 3000 cobalt workers in 1951–1982 [6]. It found a non-significant increase in mortality from lung cancer in the whole cohort (SMR 1.34, 95% CI 0.77–2.13). A significant excess mortality from lung cancer was found among workers with over ten years of employment who had died more than 20 years after the end of exposure (SMR 2.78, 95% CI 1.11–5.72). Early findings from a French study in the 1980s suggested increased mortality from cancers of the trachea, lung and bronchus among cobalt workers (SMR 4.66; 95% CI 1.46–10.64) [7]. However, the results were based on only four cases. In their follow-up study of the same population, Moulin et al. [8] could not confirm the previous results. A Norwegian study in a nickel refinery did not find any increase in risk of lung cancer from cobalt exposure [9].

The results of the studies on the carcinogenicity of cobalt vary according to the kind of industry in which the exposed workers are employed. Increased mortality from lung cancer has been found among workers in the hard metal industry [10]. In addition to cobalt, hard metal also contains tungsten carbide. According to the International Agency for Research on Cancer (IARC) the carcinogenicity of cobalt metal with tungsten carbide was evaluated as *probably* carcinogenic to humans (Group 2A), whereas cobalt metal without tungsten carbide was evaluated as *possibly* carcinogenic to humans (Group 2B). Cobalt sulphate and other soluble cobalt (II) salts were also evaluated as *possibly* carcinogenic to humans (Group 2B) [1].

In early 2014, the Netherlands Competent Authority (RIVM) notified its intention via the Registry of Intent to prepare an EU-harmonised classification and labelling proposal on ‘cobalt metal and other cobalt compounds (to be determined)’. European Commission is actively fighting against occupational cancer and aiming to amend the Carcinogens and Mutagens Directive [11]. It is important to have up-to-date scientific evidence based data as a background of legislation.

The purpose of this study was to assess the risk of cancer among workers employed in a Finnish cobalt plant, using the company’s employment records, exposure data, and data from the Finnish Cancer Registry. On the basis of this reliable data, we aimed to add to the knowledge on the carcinogenicity of cobalt without tungsten carbide in occupational settings.

## Methods

This is a retrospective cohort study. The study cohort was made up of all males employed for at least one year at the Kokkola cobalt plant (Freeport Cobalt Oy) during the period 1968–2004. The cohort of 1004 men was identified from the company’s employment records. The correct personal identity codes (PICs), vital status and possible dates of emigration or death were searched from the national Population Register Center. Nine men (0.9%) were not found in the population register and were excluded, leaving 995 workers in the final cohort (Table 1). Since 1967, all Finnish residents have had a unique PIC, which is used in all main registers in Finland. The PIC enables reliable automatic record linkage.

**Table 1:** Number of male workers (N) in cobalt plant cohort, and person-years during 1969–2013, by age and exposure group (for definitions, see Table 2). The numbers in the N column in different age groups refer to the age at the beginning of follow-up

	N	Person-years
Total	995	26,083.2
High exposure	380	11,254.9
Age (years)		
15–29	269	1480.0
30–44	103	4243.6
45–59	7	3663.5
60–74	1	1736.5
75+	-	131.6
Moderate exposure	159	2823.4
Age (years)		
15–29	122	563.4
30–44	35	1378.4
45–59	2	674.8
60–74	-	205.8
75+	-	1.0
Low exposure	364	9966.7
Age (years)		
15–29	275	1431.2
30–44	81	3903.3
45–59	8	2987.7
60–74	-	1522.6
75+	-	121.9
Variable exposure with peak exposures	110	2174.1
Age (years)		
15–29	94	475.9
30–44	16	960.6
45–59	-	551.2
60–74	-	182.8
75+	-	3.7

We used PICs as keys in the follow-up for cancer through the files of the population-based nationwide Finnish Cancer Registry. Follow-up began on the date when the person had been working for one year at the Kokkola cobalt plant, and ended at emigration, death or on 31 December 2013, whichever came first. We also calculated the cancer risk as starting from the date when a person had worked for five years at the cobalt plant.

The numbers of observed cases and person-years at risk were counted, in five-year age groups and five-year calendar periods. The expected numbers of cases for total cancer and for specific cancer types were calculated by multiplying the number of person-years in each stratum by the corresponding cancer incidence among men in the central hospital catchment area of Central Ostrobothnia around the Kokkola cobalt plant.

To calculate the standardised incidence ratio (SIR), the observed number of cases was divided by the expected number. The 95% confidence interval (95% CI) for the SIR was based on the assumption that the number of observed cases followed a Poisson distribution.

A non-commercial software program of Finnish Cancer Registry was used for statistical analysis. We call an SIR as “statistically significant” if its 95% confidence interval does not include value 1.0.

### Workplace exposures

**Fig. 1:**
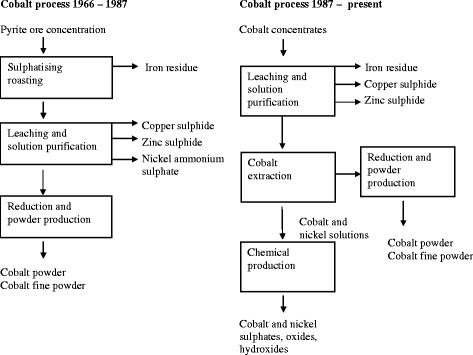
Cobalt production process in the Kokkola cobalt plant in 1966 – 1987 and 1987 – present

Between 1966 and 1987, cobalt powder was produced from pyrite ore concentrate at the Kokkola cobalt plant. After 1987, cobalt powder, inorganic cobalt, and nickel compounds have been produced using by-products of the metallurgic industry as raw material (Fig. 1).

In sulphatising roasting, dust in the ambient air was found to contain 15–20% iron, 1% zinc, 0.4% cobalt, and 0.2% nickel, whereas in leaching building, the dust consisted of metal sulphides and sulphates. The highest exposure levels of nickel (0.12 mg/m^3^) were measured in the chemical department during 1987–1999, otherwise exposure levels have been ≤0.04 mg/m^3^. Cobalt and nickel were present as water-soluble sulphates. In the reduction plant and powder production facility, cobalt is mainly in the form of cobalt powder and fine powder. In the chemical department, the cobalt and nickel compounds were mainly sulphates, carbonates, oxides, and hydroxides.

**Fig. 2:**
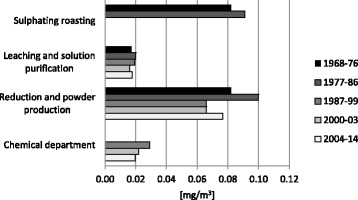
Mean cobalt exposure levels at Kokkola cobalt plant in 1968–2014

Total exposure to dust, cobalt, nickel, sulphur dioxide, hydrogen sulphide, and ammonia has been regularly monitored several times a year since 1966, as both stationary measurements and personal samples. The mean exposure level of total dust was high in the sulphatising roasting department, at 8.5 mg/m^3^. The mean levels of cobalt in the workplace air in 1967–2003 are presented in Fig. 2. The methods of measuring workplace exposures have been described in detail earlier [12, 13].

According to biological monitoring surveillance, the highest exposure to cobalt was in the reduction and powder production department. The highest urinary content of cobalt was about 16,000 nmol/l (level of unexposed persons being <40 nmol/l). In the solution purification and chemical departments, the urinary cobalt levels were between 200 and 2000 nmol/l. Respirators were available since the plant started operating, and became mandatory in the last ten years in the powder production and chemical departments. The biological monitoring results show that exposure is still considerable, despite the intensified use of respirators [12, 13].

### Exposure groups

The cohort was divided into subcohorts by exposure levels, according to the department in which they had started working during their employment at the plant (Tables 1, 2). The exposure in different departments was classified according to the industrial hygienic measurements (Fig. 2) and biological monitoring. Exposure in factory maintenance was classified as variable, because it includes e.g. repairs in different departments with possible peak exposures when the machinery is out of order.

**Table 2:** Exposure groups according to departments

Definition	Departments
Variable exposure with peak exposures	Factory maintenance
Low exposure	Leaching and solution purification
Moderate exposure	Chemical department, test plant
High exposure	Sulphatising roasting, reduction and powder production

## Results

The follow-up cohort consisted of 995 men with 26,083 person-years (Table 1). The mean follow-up of a person was thus 26.2 years.

During the follow-up period, 92 cases of cancer were diagnosed, and the expected number was 91.9 (SIR 1.00, 95% CI 0.81–1.22; Table 3). Five cases were observed 5–15 years after the beginning of follow-up and 87 cases more than 15 years after the beginning of follow-up.

**Table 3:** Observed (Obs) and expected (Exp) numbers of cancer cases and standardised incidence ratios (SIR) with 95% confidence intervals (CI) among male workers at the Kokkola cobalt plant during 1969–2013, by site and duration of employment. Only primary sites with ≥2 observed or expected cancer cases included

Primary site	Employment >1 year				Employment >5 years			
	Obs	Exp	SIR	95% CI	Obs	Exp	SIR	95% CI
All sites	92	91.9	1.00	0.81–1.22	77	71.6	1.08	0.85–1.34
Tongue	3	0.41	7.39	1.52–21.6	3	0.30	10.0	2.06–29.2
Oesophagus	2	1.15	1.74	0.21–6.28	2	0.89	2.24	0.27–8.08
Stomach	7	3.47	2.01	0.81–4.15	5	2.73	1.83	0.59–4.26
Colon	4	4.36	0.92	0.25–2.34	4	3.46	1.16	0.32–2.96
Rectum, rectosigmoid	4	3.80	1.05	0.29–2.69	3	2.93	1.03	0.21–2.99
Pancreas	2	3.44	0.58	0.07–2.09	1	2.69	0.37	0.01–2.07
Larynx, epiglottis	2	0.81	2.45	0.30–8.86	2	0.65	3.09	0.37–11.2
Lung, trachea	6	12.0	0.50	0.18–1.08	5	9.55	0.52	0.17–1.22
Melanoma of the skin	1	3.29	0.30	0.01–1.69	1	2.53	0.39	0.01–2.20
Skin, non-melanoma	3	2.78	1.08	0.22–3.15	3	2.22	1.35	0.28–3.94
Basal cell carcinoma of the skin	18	19.2	0.94	0.56–1.48	12	15.1	0.80	0.41–1.38
Prostate	33	24.4	1.35	0.93–1.89	26	19.4	1.34	0.87–1.96
Kidney	2	3.81	0.52	0.06–1.89	2	3.00	0.67	0.08–2.40
Bladder and urinary tract	9	4.79	1.88	0.86–3.56	6	3.76	1.60	0.59–3.47
Brain, central nervous system	2	2.82	0.71	0.09–2.56	2	2.07	0.97	0.12–3.49
Thyroid gland	2	0.99	2.01	0.24–7.26	2	0.72	2.78	0.34–10.0
Non-Hodgkin lymphoma	3	4.44	0.68	0.14–1.97	3	3.42	0.88	0.18–2.56
Leukaemia	3	2.11	1.42	0.29–4.15	3	1.58	1.90	0.39–5.54

A total of 77 cases of cancer were diagnosed among men who had been working in the plant for over five years (SIR 1.08, 95% CI 0.85–1.34) (Table 3). The overall cancer incidence was not significantly elevated in any of the exposure groups (Table 4).

**Table 4:** Observed (Obs) and expected (Exp) numbers of cancer cases (all sites, lung and nasal cancer) and standardised incidence ratios (SIR) with 95% CI during 1967–2011 among workers in the Kokkola cobalt plant during 1969–2013with employment of >1 year, by exposure group

Exposure group	All sites				Lung, trachea				Tongue				Bladder			
	Obs	Exp	SIR	95% CI	Obs	Exp	SIR	95% CI	Obs	Exp	SIR	95% CI	Obs	Exp	SIR	95% CI
Variable	7	5.02	1.39	0.56–2.87	-	0.55	0.00	0.00–6.68	1	0.04	26.4	0.67–14.0	-	0.25	0.00	0.00–15.0
Low	42	37.8	1.11	0.80–1.50	2	4.89	0.41	0.05–1.47	1	0.15	6.48	0.16–36.1	6	1.96	3.07	1.12–6.67
Moderate	4	6.02	0.66	0.18–1.70	-	0.66	0.00	0.00–5.56	-	0.06	0.00	0.00	-	0.30	0.00	0.00–12.2
High	39	43.6	0.90	0.64–1.22	4	5.95	0.67	0.18–1.72	1	0.16	6.12	0.15–34.1	3	2.32	1.30	0.27–3.78
Total	92	91.9	1.00	0.81–1.22	6	12.0	0.50	0.18–1.08	3	0.41	7.39	1.52–21.6	9	4.79	1.88	0.86–3.56

The SIR for lung cancer among men with at least one year of employment was 0.50 (95% CI 0.18–1.08) and among men with at least five years of employment 0.52 (95%CI 0.17–1.22) (Table 3). None of the exposure group-specific SIRs for lung cancer was significantly different from 1.0 (Table 4). Three of the lung cancer cases were in the age group 45–59 years and three in the age group 60–74 years. The age specific SIRs for lung cancer did not differ significantly from those of the reference population (age group 45–59 years: SIR 0.75, 95% CI 0.15–2.18 and age group 60–74 years: SIR 0.42, 95% CI 0.09–1.24).

The incidence of tongue cancer was significantly increased (SIR 7.39; 95% CI 1.52–21.6) (Table 3). The age of the patients at the time of diagnosis was 39, 52 and 70 years. The age group specific SIRs for tongue cancer did not differ significantly from those of the reference population (age group 30–44 years: SIR 16.35, 95% CI 0.41–91.08; age group 45–59 years: SIR 8.02, 95% CI 0.20–44.66 and age group 60–74 years: SIR 16.23, 95% CI 0.41–90.40). All three cases were smokers. One of them had worked for 20.5 years in the cobalt plant (variable exposure group), one had worked there for 36.1 years (low exposure group) and the third for 15.8 years (high exposure group).

There was an excess of bladder cancer cases in the low exposure group (SIR 3.07; 95% CI 1.12–6.67) (Table 4). One bladder cancer patient was a non-smoker, four were smokers and the smoking status of four patients was unknown. The SIRs for stomach cancer, larynx cancer and thyroid cancer exceeded 2.0 but were based on small numbers of cases and were not statistically significant (Table 3).

## Discussion

Early findings from a French study in the 1980s suggested that cobalt workers may be at an increased risk of cancers of the trachea, lung and bronchus [7]. Later findings indicated that occupational exposure to cobalt in the ambient air does not increase either the total cancer risk or the risk of lung cancer [8]. The results of the present study are in line with the no-increase findings. In the present study of a cohort of 995 men working in a cobalt plant, with a mean follow-up of 26.2 years, we observed no elevated overall cancer incidence or elevated incidence of lung cancer.

Our cohort consisted of all employees who had been working in the integrated production unit of the Kokkola cobalt plant during 1969–2004. Identification of cohort members and follow-up for deaths and emigration were complete for the period of this study. The completeness of cancer registration in Finland is at least 99% [14], and the computerised record linkage procedure precise [15]. Therefore, technical incompleteness does not cause bias in the results.

### Exposure assessment

The mean levels of cobalt in the ambient air in this plant were generally at the level of the current occupational exposure limit in Finland (0.02 mg/m^3^). During the first years of cobalt production, the cobalt levels may have been considerably higher, over 1 mg/m^3^, especially in the roasting department. The Finnish occupational limits were also often exceeded in the reduction and powder production departments.

Cobalt exposure was monitored with exceptional accuracy from the early days of the studied plant. The working history of the patients was verified from the registers, which minimises recall and information bias. This was not the case in previous epidemiological studies, which did not report either the exposure levels of cobalt or simultaneous co-exposures [7].

Of the other simultaneous co-exposures, nickel is considered as carcinogenic. There has been nickel exposure in leaching, solution, purification and chemical departments. In a recent study on cancer risk in a Finnish nickel refinery the increased lung cancer and sinonasal cancer risk was found in the most nickel-exposed work site where the nickel concentration had been ≥0.2 mg/m^3^ [16]. The nickel exposure levels have been much lower in the cobalt plant than in the nickel refinery, and in the present study there were no sinonasal cancers and the risk of lung cancer was low.

### SIR

The previous epidemiological studies on the carcinogenicity of cobalt used the standardised mortality ratio (SMR) as a measure of cancer risk, comparing the number of deaths in the cohort with the expected number of deaths, calculated from the mortality rates of the general population [7, 8, 10]. As the majority of cancer patients die from non-cancer causes of death, the SIR is a more sensitive way of analysing the effects of cobalt than the SMR.

### Overall cancer

The overall cancer incidence was not elevated in the present study. We used the incidence rates of the population of Central Ostrobothnia Finland as the main reference, because incidence rates vary geographically. Cancer incidence also varies according to socioeconomic position. The majority of the workers in the departments of the Kokkola cobalt plant were skilled blue-collar workers, whereas the reference group included all socioeconomic groups. It is known that the cancer incidence of blue-collar workers in most cancer types is close to the population average [17], and hence, the reference rates used in our calculations of expected numbers of cases should be valid.

Healthy worker effect is a phenomenon initially observed in studies of occupational diseases: workers usually exhibit lower overall death rates than the general population because the severely ill and chronically disabled are ordinarily excluded from employment [18]. The healthy worker effect might also affect cancer incidence in the first years after employment but the effect is much smaller than it would be in a study on mortality. In the Kokkola cobalt plant there was no selection of workers because of possible cancer risk. No markers or tests were used in pre-employment health examinations of the plant to exclude individuals that could be in risk of cancer. We could follow the workers also after the end of employment. Thus, if they had to leave work because of health reasons, they were still included in our cohort. Therefore, we suggest that healthy worker effect did not play a marked role in this study.

A dose-response relationship between exposure level of cobalt and cancer incidence would have supported the idea of cobalt as a causative agent of the cancers. However, the overall cancer incidence did not increase according to the cobalt exposure level.

### Lung cancer

In the present study, the incidence of lung cancer cases decreased by 50%, and even more among the cohort members who had been working in the same department for more than five years.

A French study [7] found an increased SMR for lung cancer among cobalt workers, but a follow-up study of the same cohort with an extended observation period could not verify the previous results [8]. In that French study, the exposing compounds were quite comparable to those in our study, but the exposure levels were not known.

### Tongue cancer

The total study group contained three cases of tongue cancer. The SIR for tongue cancer was significantly higher in the total group (7.39, 95% CI 1.52–21.60). In different exposure or age groups the increase was not statistically significant. To our knowledge, no previous studies have associated cobalt exposure with tongue cancer. According to the literature, the most significant risk factors for oral cancer are smoking and alcohol use [19, 20]. All three cases of tongue cancer in our cohort were smokers. We do not have data on their alcohol consumption.

Interaction of different carcinogenic metals could be a hypothetical explanation for the excess of tongue cancers. However, none of the tongue cancer patients had worked in the chemical department where the nickel exposure was highest, but tobacco smoke contains also different metals including aluminium, cadmium, chromium, nickel, lead, mercury, selenium, vanadium, manganese and zinc [21], which may have synergistic effects with cobalt exposure. Metals are thought to promote cancer by a number of common mechanisms. In terms of direct damage to DNA, most metals are only weakly mutagenic; however, many are strong co-carcinogens, promoting a synergistic effect in the presence of other cancer-causing agents [22]. Because the small number of tongue cases here, the excess may be explained by chance alone.

### Cancer of urinary bladder

Nine cases were bladder cancer, which is nearly twice the expected number, but the difference was not statistically significant. However, the lowest exposure group had a statistically significant three-fold excess of urinary bladder cancer cases. All in all, 2/3 of the cases were from the lowest exposure category. The most important risk factor for bladder cancer is tobacco smoking [23]. Only one bladder cancer patient was a non-smoker out of five cases with known smoking status. Occupationally, aromatic amines are known to cause bladder cancer as an occupational disease [24]. No one in this study group was known to have been exposed to aromatic amines.

### Smoking

The most important source of incomparability between the results derived from different cohorts is usually confounding due to smoking, which has not been controlled for in most studies and which may lead to a bias in a different direction, depending on whether smoking in the cohort is lower or higher than in the reference population. According to data gathered in 2000, the prevalence of current smokers among the employees of the Kokkola cobalt plant was 31.8% [13]. The prevalence of daily smokers in the male population in the province of Central Ostrobothnia around the cobalt plant in 1990–2005 varied from 18% in the highest educational class to 25% in the lowest [25]. These percentages may be an underestimation of smokers, possibly due to the small sample size. During this same time period, the prevalence of daily smokers in the whole country was about 30% [25]. Moreover, daily smokers and current smokers may represent different groups. Hence, the explanation of the SIR 0.5 for lung cancer in our cohort is apparently not a lower smoking prevalence as compared to the reference population.

### Different effects of different compounds

An assessment of cobalt and its compounds requires a clear distinction between different compounds and needs to take into account the different mechanisms involved. There is evidence that soluble cobalt (II) cations exert genotoxic and carcinogenic activity both in vitro and in vivo in experimental systems [26, 27]. There is also evidence that hard metal particles that contain tungsten carbide, in addition to cobalt, exert genotoxic and carcinogenic activity in vitro and in human studies [28]. However, the conclusion of IARC (2006) is that the evidence is limited in humans for increased risk of lung cancer in case of cobalt with tungsten carbide and inadequate in case of cobalt without tungsten carbide [1]. For carcinogenicity of cobalt oxides and other compounds there is insufficient information [28].

## Conclusions

The results suggest that occupational exposure to cobalt is not associated with an increased overall cancer risk or lung cancer risk among cobalt workers. Unexpectedly we found a significant increase in the incidence of tongue cancer. There are no previous data from either animal or human studies to support an association between cobalt and tongue cancer. Because of the small number of cancer cases the results must be interpreted with caution.

## Abbreviations

CI: Confidence interval
IARC: International Agency for Research on Cancer
SIR: Standardised incidence ratio
